# Personalized and Precision Medicine 2022

**DOI:** 10.3390/jpm13030459

**Published:** 2023-03-01

**Authors:** Anne-Marie Caminade

**Affiliations:** 1 Laboratoire de Chimie de Coordination (LCC), CNRS UPR8241, 205 Route de Narbonne, CEDEX 4, 31077 Toulouse, France; anne-marie.caminade@lcc-toulouse.fr; 2 LCC-CNRS, Université de Toulouse, CNRS, Toulouse, France

This Special Issue, “Personalized and Precision Medicine 2022” (https://www.mdpi.com/journal/jpm/special_issues/pemed_2021 (accessed on 24 February 2023)), in the *Journal of Personalized Medicine*, was first proposed at the International Conference on Personalized and Precision Medicine PEMED 2021, which occurred online on 7–9 April 2021 (https://premc.org/conferences/pemed-personalized-precision-medicine/ (accessed on 24 February 2023)), after three editions organized in Paris (25–27 June 2018), Barcelona (15–17 May 2019), and Munich (19–21 February 2020). The diversity of studies carried out in connection with personalized and precision medicine was clearly emphasized during these international conferences, as also observed in the 20 papers of this Special Issue. The most represented topics concern brain diseases, cancers, lungs obstruction, muscles, and skeletal diseases. Other specific topics include the intestinal microbiome or COVID-19, emerging methods for imaging and using radiotherapy, and a promising new class of therapeutic molecules known as dendrimers. 

Alzheimer’s disease is the most prevalent brain disease and can take several clinical presentations. The article by V. Bessi et al. [1] compares the empathy deficit and its neural basis for Alzheimer’s patients with either logopenic primary progressive aphasia or amnesia. Metabolic disfunctions were observed in different brain regions, depending on the type of the Alzheimer’s disease, but they induce the same damage of cognitive empathy and personal distress over time. Contrary to Alzheimer’s disease, Huntington’s disease has a low prevalence, but it causes a long-lasting burden in affected families. The article by K. Hubčíková et al. [2] discusses the psychosocial impact of this disease on the affected families in the Slowak Republic. Comprehensive genetic counselling, including the possibility of preimplantation genetic diagnosis, can particularly mitigate the psychosocial effects and burden induced by Huntington’s disease. Multiple sclerosis is a chronic inflammatory and neurodegenerative disease of the central nervous system, frequently associated with multisystem comorbidities. The review by V. Nocity et al. [3] summarizes the available data on the incidence and prevalence of autoimmune diseases in multiple sclerosis, their effect on the clinical course of the disease, and their impact on the treatment choice.

Spastic-type cerebral palsy is a brain disease that induces a complex neuromuscular disorder, involving altered skeletal muscle microanatomy and growth. The paper by R.E. Akins et al. [4] focuses on the poorly studied mechanisms that contribute to muscle pathophysiology and dysfunction. They examined whether a diagnosis of spastic cerebral palsy is associated with intrinsic DNA methylation differences in myoblasts and myotubes derived from muscle-resident stem cell populations. A comparison of the skeletal muscle biopsies obtained during orthopedic surgeries between patients with and without spastic cerebral palsy demonstrated fundamental differences in DNA methylation, which may reveal new targets for studies of mechanisms that contribute to muscle dysregulation. A very different paper concerning muscles is proposed by K. Kim et al. [5], which investigates the effect of different lumbar belts in patients with nonspecific low back pain. Measurements taken by a three-dimensional motion analysis device, force plate, and surface electromyography demonstrated the positive effect of extensible lumbar belts.

Orthopedic surgeries are frequently followed by inpatient rehabilitation. The paper by D. Rak et al. [6] posed the question of comparing the efficiency between “classical” and expensive rehabilitation or fast-track rehabilitation for inpatients after total knee replacement. It is shown that one year after surgery, impatient rehabilitation does not provide long-term benefits over fast-track rehabilitation. In some cases, orthodontic malocclusion necessitates bone-anchored maxillary protraction, which induces infection or device failures that occur with conventional plates. The paper by J.-W. Kim et al. [7] displays a pilot prospective study using preoperative simulation and the 3D titanium printing of customized plates. Better results were obtained with these customized plates after two years, showing that this method is effective in treating skeletal malocclusion.

Four papers in this Special Issue are based on cancer, which is one of the leading causes of death. The paper by K.Y. Arga and R. Sinha et al. [8] explores cancer hallmark proteins in different cancer types, studied with the aim of discovering measurable indicators. A pan-cancer analysis to map differentially interacting hallmarks of cancer proteins associated with 12 common cancers was carried out. The study presents candidate systems’ biomarkers that may be valuable for improving personalized treatment strategies for various cancers. The review by K. Kodama et al. [9] proposes a large overview of the discovery and development of 107 anticancer drugs, in connection with interorganizational collaboration, from 1998 to 2018. It is shown that immune checkpoint blockade agents are a significantly active area in interorganizational transactions, suggesting that such types of agents are a paradigm for cancer treatment, resulting in huge product sales and continuous indication expansion.

Two papers from the same group focus on the treatment of bladder cancers. The first of the two, by A. Sherif et al. [10], uses a clinical multicenter database to demonstrate that a high aspartate transaminase–alanine aminotransferase ratio (De Ritis ratio) before treatment of muscle invasive bladder cancer is associated with increased mortality. However, this ratio cannot be used for downstaging prediction. The second paper by A. Sherif et al. [11] investigates thromboembolic events in patients with muscle-invasive bladder cancer undergoing neoadjuvant chemotherapy. It was suggested that low-molecular-weight heparin is a possible prophylaxis, but a high incidence of decreased renal function was observed in these patients. Amongst the neoadjuvant-chemotherapy-administered patients with thromboembolic events, 41% of patients had decreased renal function, thus, reducing the likelihood of them benefitting from low-molecular-weight heparin prophylaxis.

The review by S. Boussios et al. [12] focuses on three massive healthcare threats (cancer, mucormycosis, and COVID-19), and the danger of one of these diseases becoming predisposed to another. The conclusion was that COVID-19 and mucormycosis pose a larger risk in cancer patients.

Besides the COVID-19 virus, various other obstructive lung diseases are known. The paper by P. Steiropoulos et al. [13] evaluates vitamin D serum levels in patients with obstructive sleep apnea, eventually associated with chronic obstructive pulmonary disease (such an association is defined as the overlap syndrome). Lower vitamin D levels have been observed in patients with overlap syndrome compared with obstructive sleep apnea patients and non-apneic controls, which might be associated with disease severity. Another paper by D.-K. Kim et al. [14] on patients with obstructive sleep apnea proposes an ultra-short-term analysis to detect the balance of autonomic nervous system activity. The overall heart rate variability feature alterations obtained by this method indicated sympathetic overactivity dependent on obstructive sleep apnea severity. The risk factors of mortality in patients with carbapenem-resistant *Klebsiella pneumoniae* bloodstream infection are analyzed in the paper by M.-C. Lu et al. [15]. No effective antimicrobial regimen could be identified, but diverse risk factors were identified, such as lower platelet count and a higher Pitt bacteremia score.

For instance, miscellaneous topics concern the prediction of hepatotoxicity in azathioprine-treated patients with auto-immune diseases. The paper by W.T. Hung et al. [16] studies in Asian patients with thiopurine methyltransferase, which is a rate-limiting enzyme in azathioprine metabolization. Genetic variants in the thiopurine methyltransferase were analyzed, showing that the non-normal metabolizers were associated with hepatotoxicity. Another example discusses the intestinal microbiome. The paper by A.R. Moschen et al. [17] focuses on the short- and long-term effects of capsules of a purified extract from the European black elderberries on the microbiome composition. The supplementation was well tolerated, and changes in species abundance were observed over time. In particular, the relative abundance of *Akkermansia* spp., which may have beneficial effects on inflammation and metabolism, continued to increase in a subset of participants, even beyond the supplementation period.

Two different physical techniques are presented. The clinical applications of ultra-high-frequency ultrasound are proposed by D. Berritto et al. [18] for the study of many superficial targets, within the first 3 cm of skin surface. The high spatial resolution of this technic is especially suitable for innovations to diagnostic imaging of hands, wrists, and feet. A review of the modern approaches of non-oncological radiotherapy is proposed by V. Nardone et al. [19]. Different disorders such as heart tachycardia, soft tissue disorders, muscle–skeletal disorders, osteoarthritis and osteoarthrosis, neurological disorders, and Graves’ ophthalmopathy are considered.

Finally, an emerging family of macromolecules with potential therapeutic properties, known as dendrimers, are reviewed by A.-M. Caminade [20], with emphasis on their clinical trials. Many of these clinical trials have recently been reported (2020–2022), with the aim of treating essentially bacterial vaginosis, cancers, and COVID-19.

Overall, the twenty papers in this Special Issue constitute an impressionist overview of the state-of-the-art research in the field of personalized and precision medicine, with some perspectives on the future of this topic.

## References

[1] Giacomucci G., Polito C., Berti V., Padiglioni S., Galdo G., Mazzeo S., Bergamin E., Moschini V., Morinelli C., Nuti C. (2023). Differences and Similarities in Empathy Deficit and Its Neural Basis between Logopenic and Amnesic Alzheimer’s Disease. J. Pers. Med..

[2] Hubčíková K., Rakús T., Mühlbäck A., Benetin J., Bruncvik L., Petrášová Z., Bušková J., Brunovský M. (2022). Psychosocial Impact of Huntington’s Disease and Incentives to Improve Care for Affected Families in the Underserved Region of the Slovak Republic. J. Pers. Med..

[3] Nociti V., Romozzi M. (2022). Multiple Sclerosis and Autoimmune Comorbidities. J. Pers. Med..

[4] Robinson K.G., Marsh A.G., Lee S.K., Hicks J., Romero B., Batish M., Crowgey E.L., Shrader M.W., Akins R.E. (2022). DNA Methylation Analysis Reveals Distinct Patterns in Satellite Cell–Derived Myogenic Progenitor Cells of Subjects with Spastic Cerebral Palsy. J. Pers. Med..

[5] Im S.-C., Seo S.-W., Kang N.-Y., Jo H., Kim K. (2022). The Effect of Lumbar Belts with Different Extensibilities on Kinematic, Kinetic, and Muscle Activity of Sit-to-Stand Motions in Patients with Nonspecific Low Back Pain. J. Pers. Med..

[6] Rak D., Nedopil A.J., Sayre E.C., Masri B.A., Rudert M. (2022). Postoperative Inpatient Rehabilitation Does Not Increase Knee Function after Primary Total Knee Arthroplasty. J. Pers. Med..

[7] Kim M., Li J., Kim S., Kim W., Kim S.-H., Lee S.-M., Park Y.L., Yang S., Kim J.-W. (2021). Individualized 3D-Printed Bone-Anchored Maxillary Protraction Device for Growth Modification in Skeletal Class III Malocclusion. J. Pers. Med..

[8] Kori M., Ozdemir G.E., Arga K.Y., Sinha R. (2022). A Pan-Cancer Atlas of Differentially Interacting Hallmarks of Cancer Proteins. J. Pers. Med..

[9] Djurian A., Makino T., Lim Y., Sengoku S., Kodama K. (2021). Dynamic Collaborations for the Development of Immune Checkpoint Blockade Agents. J. Pers. Med..

[10] Eriksson V., Holmkvist O., Huge Y., Johansson M., Alamdari F., Svensson J., Aljabery F., Sherif A. (2022). A Retrospective Analysis of the De Ritis Ratio in Muscle Invasive Bladder Cancer, with Focus on Tumor Response and Long-Term Survival in Patients Receiving Neoadjuvant Chemotherapy and in Chemo Naïve Cystectomy Patients—A Study of a Clinical Multicentre Database. J. Pers. Med..

[11] Rydell H., Ericson A., Eriksson V., Johansson M., Svensson J., Banday V., Sherif A. (2022). Thromboembolic Events in Patients Undergoing Neoadjuvant Chemotherapy and Radical Cystectomy for Muscle-Invasive Bladder Cancer: A Study of Renal Impairment in Relation to Potential Thromboprophylaxis. J. Pers. Med..

[12] Mahajan I., Ghose A., Gupta D., Manasvi M., Bhandari S., Das A., Sanchez E., Boussios S. (2022). COVID-19, Mucormycosis and Cancer: The Triple Threat—Hypothesis or Reality?. J. Pers. Med..

[13] Archontogeorgis K., Voulgaris A., Nena E., Zissimopoulos A., Bouloukaki I., Schiza S.E., Steiropoulos P. (2022). Vitamin D Levels in Patients with Overlap Syndrome, Is It Associated with Disease Severity?. J. Pers. Med..

[14] Ha S.-S., Kim D.-K. (2022). Diagnostic Efficacy of Ultra-Short Term HRV Analysis in Obstructive Sleep Apnea. J. Pers. Med..

[15] Liu K.-S., Tong Y.-S., Lee M.-T., Lin H.-Y., Lu M.-C. (2021). Risk Factors of 30-Day All-Cause Mortality in Patients with Carbapenem-Resistant *Klebsiella pneumoniae* Bloodstream Infection. J. Pers. Med..

[16] Sheu H.-S., Chen Y.-M., Liao Y.-J., Wei C.-Y., Chen J.-P., Lin H.-J., Hung W.-T., Huang W.-N., Chen Y.-H. (2022). Thiopurine S-Methyltransferase Polymorphisms Predict Hepatotoxicity in Azathioprine-Treated Patients with Autoimmune Diseases. J. Pers. Med..

[17] Reider S., Watschinger C., Längle J., Pachmann U., Przysiecki N., Pfister A., Zollner A., Tilg H., Plattner S., Moschen A.R. (2022). Short- and Long-Term Effects of a Prebiotic Intervention with Polyphenols Extracted from European Black Elderberry—Sustained Expansion of *Akkermansia* spp.. J. Pers. Med..

[18] Russo A., Reginelli A., Lacasella G.V., Grassi E., Karaboue M.A.A., Quarto T., Busetto G.M., Aliprandi A., Grassi R., Berritto D. (2022). Clinical Application of Ultra-High-Frequency Ultrasound. J. Pers. Med..

[19] Nardone V., D’Ippolito E., Grassi R., Sangiovanni A., Gagliardi F., De Marco G., Menditti V.S., D’Ambrosio L., Cioce F., Boldrini L. (2022). Non-Oncological Radiotherapy: A Review of Modern Approaches. J. Pers. Med..

[20] Caminade A.-M. (2022). Dendrimers, an Emerging Opportunity in Personalized Medicine?. J. Pers. Med..

